# Late effects in survivors of infantile acute leukemia: a study of the L.E.A program

**DOI:** 10.1038/bcj.2016.129

**Published:** 2017-01-20

**Authors:** V Gandemer, J Bonneau, C Oudin, J Berbis, Y Bertrand, M-D Tabone, S Ducassou, P Chastagner, B Brethon, J-H Dalle, S Thouvenin, M Poiree, D Plantaz, J Kanold, N Sirvent, P Lutz, Z Hamidou, A Baruchel, G Leverger, P Auquier, G Michel

**Affiliations:** 1 Department of Pediatric Onco-Hematology, University Hospital of Rennes, Rennes1 University, Rennes, France; 2 Department of Pediatric Hematology and Oncology, La Timone Children's Hospital, APHM and Aix-Marseille University, Marseille, France; 3 EA3279, Self-perceived Health Assessment Research Unit, School of Medicine, Aix-Marseilles University, Marseilles, France; 4 Department of Pediatric Hematology, Institut d'Hématologie et Oncologie Pédiatrique, Claude Bernard University Lyon, Paris, France; 5 Department of Pediatric Onco-Hematology, APHP, GHUEP, Armand Trousseau Hospital, Paris, France; 6 Department of Pediatric Onco-Hematology, University Hospital of Bordeaux, Bordeaux, France; 7 Department of Pediatric Onco-Hematology, University Hospital of Nancy, Nancy, France; 8 Department of Pediatric Hematology, APHP, Robert Debre Hospital, Paris Diderot University, Paris, France; 9 Department of Pediatric Onco-Hematology, University Hospital of Saint-Etienne, Saint-Etienne, France; 10 Department of Pediatric Onco-Hematology, University Hospital of Nice, Nice, France; 11 Department of Pediatric Onco-Hematology, University Hospital of Grenoble, Grenoble, France; 12 Department of Pediatric Onco-Hematology, CIC Inserm 501, CHU Clermont-Ferrand, Clermont-Ferrand, France; 13 Department of Pediatric Onco-Hematology, University Hospital of Montpellier, Montpellier, France; 14 Department of Pediatric Onco-Hematology, University Hospital of Strasbourg, Strasbourg, France

Studies of long-term survivors of childhood leukemia have shown that many patients have significant late effects due to their treatment.^1^ Because of the rarity and poor prognosis of infant leukemia,^2, 3, 4, 5^ few papers on the late effects in this population have been published, whereas the use of maximally intensified therapies to improve survival rates and the vulnerability of these young children to complications and acute toxicity present major challenges.^6^ We examined a cohort of long-term survivors included in the Leucémies de l'Enfant et de l'Adolescent program (L.E.A.) who were <1 year of age at diagnosis without any Down syndrome or other congenital genetic abnormalities and studied the influence of age in months, sex, the type of leukemia and the use of hematopoietic stem cell transplantation (HSCT) to investigate the incidence of and risk factors for late sequelae of treatment in infants. Details of the program have been previously described.^7^ All patients (or their parents) provided written informed consent. Late effects were detected by physicians through regular visits to reference centers, starting 1 year after HSCT or after completion of chemotherapy and comprised a medical examination and adequate laboratory tests. The following late effects were assessed in the entire cohort: height, weight, body mass index (BMI), gonadal function, hypothyroidism, cardiac function, iron overload, cataract, alopecia, osteonecrosis, diabetes, metabolic syndrome, central nervous system (CNS) complications and viral infection. Health Quality of Life (HQoL) of children and adolescents was assessed using the parents' version of the Vécu et Santé Perçue de l'Adolescent et l'enfant (VSP-Ap) which is completed by the parents of the children and adolescents.^8^ Adult patients were asked to complete SF-36 questionnaires.^9^

Overall, 169 patients treated in the participating centers met eligibility criteria; 113 were enrolled in the L.E.A. cohort (57 girls and 56 boys, 55 acute lymphoblastic leukemia and 58 acute myeloblastic leukemia) and all of them were included in this analysis. The absence of difference between included and not included patients is detailed in the Supplementary Table 1). At the time of this report, 55 (48.6%) patients had only one long-term follow-up visit, 28 (24.7%) were seen twice, 16 (14.1%) three times, 10 (8.8%) had four visits and four (3.5%) had five visits.

The median age at diagnosis was 6.35 months (0.03–11.94) and 23 (20.4%) were <3 months of age. The median length of follow-up was 11.71 years (1.89–34.71). Relapse occurred in 18 patients (15.9%).

Patients had been treated according to various French or European multicenter protocols depending on the period of their treatment and the type of leukemia. Sixty-three received chemotherapy only (55.8%), four (3.1%) chemotherapy and CNS irradiation, and 50 (44.2%) HSCT. CNS irradiation doses were 18 and 12 Gy, in three patients and one patient, respectively. Only three patients received total body irradiation (TBI) before HSCT: 2 at 12 Gy and one at 10 Gy. The median age at irradiation was 2.64 years (2.25–3.48) for TBI and 3.02 years (1.16–3.09) for CNS irradiation. Three boys received testis irradiation. Allogeneic transplants were performed in 41/50 (83.7%) patients and were first transplants except for three transplanted twice. The age at diagnosis for this transplanted population was ⩽6 months for 66% (*n*=33) of cases. The median follow-up was 11.55 years (1.2–24.67) from first transplant. Graft vs host disease occurred in 22 (55%) allografts and was acute for 20 (90.9%) and chronic for four (18.2%). We distinguished transplantations performed in first complete remission (CR1) (*n*=38) from those performed after CR1 (*n*=12) to focus on infancy as a risk period for long-term sequelae and to remove the impact of second line chemotherapy on late effects.

At least one late complication was observed in 84 (74.3%) of the 113 patients (Supplementary Table 2). There was a mean of 1.36 +/− 0.11 sequelae per patient for the 15 most common late complications described above (Figure 1a). The most frequent late effect was growth failure, affecting 54 patients (47.8%), 29 of them with a cumulative change in SDS⩽−2. Only one patient in that cohort was treated with growth hormone replacement therapy. Being overweight was also frequent (22.1%) as well as low weight (21.2%) and gonadal dysfunction (13.8% of the 58 assessable patients). Girls were not more frequently affected than boys (6=22.2 vs 2=6.5%, *P*=0.128) by hypogonadism and all but one received sex hormone replacement therapy. Hypothyroidism was diagnosed in 12 patients (10.6%) but was considered to be overt (elevation of thyroid stimulating hormone associated with decreased levels of free thyroxine) in only two patients (1.8%).

Univariate analysis (Supplementary Table 3) revealed that growth failure, being overweight/underweight, and hypothyroidism were significantly more frequent in children ⩽6 months of the age at diagnosis who experienced significantly more late effects with a mean of 1.66±0.18 sequelae vs 1.11±0.12 (*P*=0.01) between 6 and 12 months of age. Conversely, there was no significant difference in the occurrence of late effects according to the type of leukemia (Supplementary Table 3) or sex of the child. The frequency of patients with at least one sequela did not show a significant difference depending on whether they had an HSCT or not (74.4% of those transplanted in CR1 vs 71.4% of those who were not, *P*=0.45), but the mean number of late complications was significantly higher for transplanted children than for those who were not. This was mainly due to HSCT performed after CR1 (1.1±0.12, 1.45±0.18, 2.64±0.47 for no HSCT, HSCT in CR1 and HSCT after CR1, respectively, *P*<0.001). In contrast to HSCT performed in CR1, transplantation following a second remission increased the risk of hypogonadism (*P*=0.027), hypothyroidism (*P*=0.002), cataract (*P*=0.01) and CNS complications (*P*=0.017; Supplementary Table 4).

Multivariate analyses confirmed the protective role of older age on major (>1 SDS) growth failure (OR=0.38, *P*=0.046) and low weight (OR=0.3, *P*=0.022; Figure 1b). No impact of HSCT performed in CR1 (=CR1) was shown, whereas HSCT occurring after CR1 (=others) significantly increased the risk of hypothyroidism (OR=18.09, *P*=0.018) and CNS complications (OR=9.89, *P*=0.036; Figure 1c). In multiple linear regression analyses, the mean number of late effects experienced did not show any correlation with either HSCT in CR1 (*P*=0.277) or age at diagnosis (*P*=0.133): 0.24 (−0.19–0.68) and −0.32 (−0.744–0.099) Beta coefficient (95% CI), respectively. In contrast, HSCT performed after CR1 was associated with a significant but moderately higher sequelae risk factor (OR=1.38. *P*<0.001).

HQoL was reported by 81 parents (92% Supplementary Table 5). Scores did not show any impact of the type of leukemia, age at diagnosis or HSCT. Sex did not affect VSP-Ap subscales except for body image which was significantly lower for girls (67.5±5.1 vs 84.5±3.8, *P*=0.01). When the HRQoL of our cohort was compared with age- and sex-matched French reference scores, we found similar results (Figure 2a). Among the adults, 21/25 (84%) completed the SF-36 questionnaires (Supplementary Table 6). They reported a similar quality of life regardless of the four groups of covariates. Comparison of these results to French norms showed that the mental composite score (*P*=0.01), mental health dimension (*P*=0.02), emotional role dimension (*P*=0.001) and vitality dimension (*P*=0.01) were lower in this L.E.A group (Figure 2b) as in other reports studying HRQoL^10^ which suggests that late psychological effects are not specific to infancy.

The present report is the largest study to evaluate the long-term outcome of infantile leukemia, survivors exhibiting at least one late effect in 75% of cases dominated by growth failure and endocrine dysfunction.^6^ Those disorders are also described in the literature for survivors of overall childhood cancer and leukemia who showed a moderately increased risk for at least one chronic illness compared with siblings.^11, 12^

The frequent elimination of irradiation from recent protocols for ALL or HSCT and the restricted use of alkylating agents and anthracyclines may have contributed to the low risk for cardiac disease and CNS complications but the relatively short follow-up for some sequelae such as second tumors or metabolic syndrome, justifies continuous, long-term follow up. We demonstrated here that infants ⩽6 months of age at diagnosis require early identification and treatment of growth alterations, including hormone replacement therapy^13, 14, 15^ and that a subgroup of long-term survivors may benefit from psychological assessment and targeted intervention. We also showed that the use of HSCT for infants is feasible given the late effects in this hard to cure population. These data are important not only for designing future therapeutic regimens, but also for identifying areas for late effect surveillance and intervention.

## Figures and Tables

**Figure 1:**
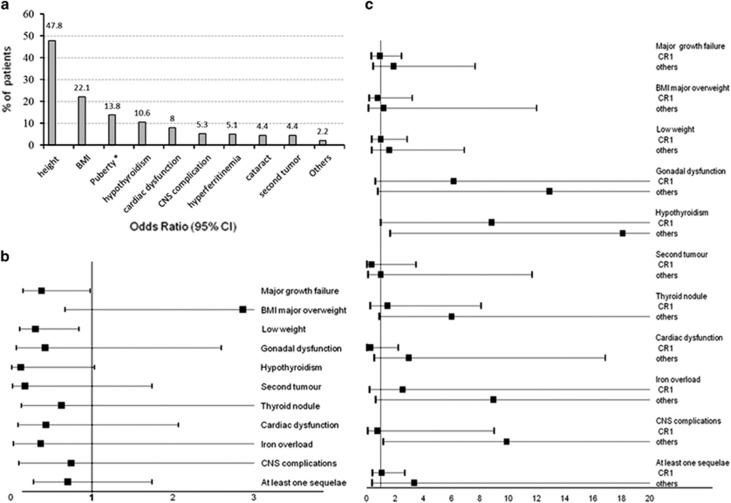
Occurrence of late effects. (**a**) in the overall population. Height, BMI, gonadal function, and thyroid function are the most frequent sequelae in our population of long term survivors of acute leukemia during infancy (<1 year). Adult height was assessable in 24 patients and revealed a median growth failure of −1.01 SDS [−5.05–1.75] that is, a final height for girls of 158 cm [140–169] and 168 cm [114–185] for boys.* Puberty was assessable in 58 patients. Precocious puberty was diagnosed in one (1.7%) patient and hypogonadism in eight (13.8%) patients. One second tumor was a thyroid malignancy, whereas nine (8%) patients presented with thyroid nodules. Central Nervous System complications consisted of seizures in three (2.7%) patients and hemiparesis, memory problems, and not-specified in one patient each (0.9%). Other complications included one case of hepatic viral transmission (0.9%) and two cases of alopecia (1.7%). There were no cases of diabetes, osteonecrosis, or metabolic syndrome (only 19 adults were assessed for the occurrence of a metabolic syndrome). Concerning fertility, three women gave birth. (**b**) Forest plot (multiple logistic regression analyses) according to age at diagnosis (six months). Age**>**6 months was significantly associated with a lower incidence of major growth failure (OR=0.38, *P*=0.046) and low weight (OR=0.3, *P*=0.022). (**c**) Forest plot according to HSCT in CR1 (= CR1) or after (= others). No impact of HSCT performed in CR1 (= CR1) was shown, whereas HSCT occurring after CR1 (=others) significantly increased the risk of hypothyroidism (OR=18.09, *P*=0.018) and CNS complications (OR=9.89, *P*=0.036). For multivariate analyses, covariates found to be significant (at a threshold of 0.05) by univariate analysis for at least one sequela were included in multiple logistic regression models. Each model was generated using its non-standardized ß-coefficient and the significance of the association set at *P*<0.05.

**Figure 2:**
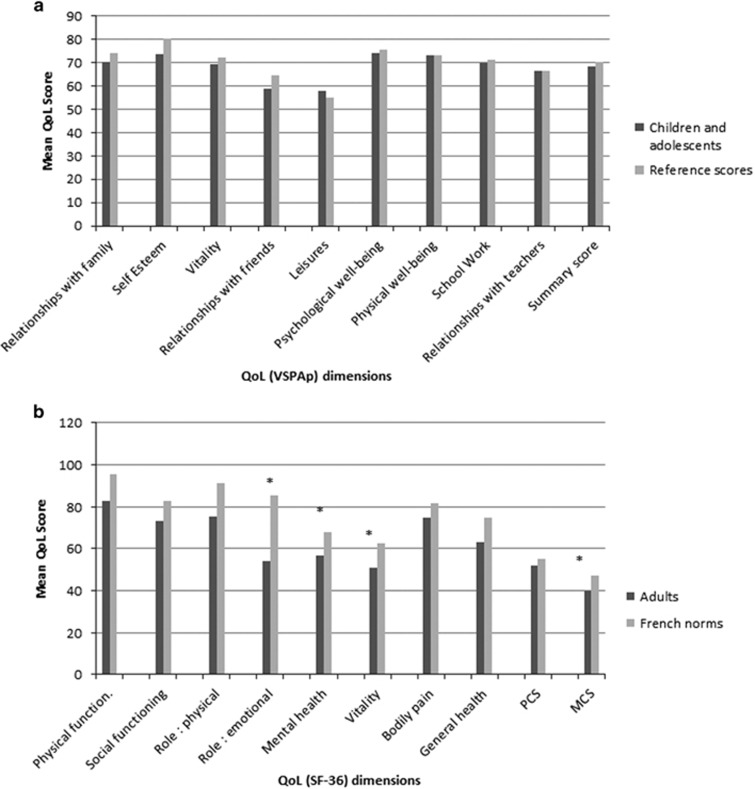
HRQoL results of the L.E.A. cohort relative to sex-and age-matched French norms (available for age >7 years) according to VSPAp (**a**) and SF-36 (**b**) questionnaires. *P<0.05 paired Student's *t*-tests for age and sex (*n*=57) with the French population reference group. PCS, Physical Composite Score; MCS, Mental Composite Score.
