# Supplementary material for: Late effects in survivors of infantile acute leukemia: a study of the L.E.A program
Source: Blood Cancer J. 2017 Jan 20;7(1):e518–. doi: 10.1038/bcj.2016.129 (PMC5301036; doi:10.1038/bcj.2016.129)
Supplement: Supplementary Tables [file bcj2016129x1.docx]

Chi2 or Fisher exact tests were used to compare qualitative variables. Quantitative variables were compared using the Student *t* test or the Mann-Whitney test

Supplemental Table 1: Characteristics of eligible children according to inclusion in the program L.E.A

| n (%) | **Enrolled children n=113 (66.9%)** | **Non enrolled children**  **n=56 (33.1%)** | **p** |
| --- | --- | --- | --- |
| *Sex*  Girls  Boys | 57 (74.0)  56 (64.4) | 20 (26.0)  31 (35.6) | 0.18 |
| *Age at diagnosis*  < 3 months   - 1. months   >6 months | 23 (69.7)  30 (62.5)  60 (68.2) | 10 (30.3)  18 (37.5)  28 (31.8) | 0.74 |
| mean (years *± SEM)* | 6.3 *±* 0.3 | 6.4 *±* 0.5 | 0.79 |
| *Date of diagnosis (divided into quartiles)*  20-Apr-1980 - 13-Nov-1996  14-Nov-1996 - 13-Aug-2001  14-Aug-2001 - 07-Jun-2007  08-Jun-2007 - 15-Oct-2011 | 29 (61.7)  28 (71.8)  28 (71.8)  28 (63.6) | 18 (38.3)  11 (28.2)  11 (28.2)  16 (36.4) | 0.65 |
| *Type of leukemia*  ALL  AML | 55 (64.0)  58 (69.9) | 31 (36.0)  25 (30.1) | 0.41 |
| *Transplantation*  No  Yes | 63 (67.0)  50 (67.6) | 31 (33.0)  24 (32.4) | 0.94 |
| *Relapse*  No  Yes | 95 (70.4)  18 (52.9) | 40 (29.6)  16 (47.1) | 0.06 |

AL of ambiguous lineage were grouped with AML

Supplemental Table 2: Patients clinical characteristics according to the occurrence of late effects.

|  | | **≥ 1 late effect(s)** | |  | | |
| --- | --- | --- | --- | --- | --- | --- |
| **Number of patients (%)** | | | **Yes** | | **No** | **p-value** |
| **n=113** | | | **84(74.3)** | | **29 (25.7)** |  |
| **Age** |  | |  | |  |  |
| < 3 months | 23(20.4) | | 17 (20.2) | | 6 (20.7) | 0.39 |
| 3-6 months | 30(26.5) | | 25 (29.8) | | 5 (17.2) |  |
| ≥ 6 months | 60(53.1) | | 42 (50.0) | | 18 (62.1) |  |
| **Transplantation history** |  | |  | |  |  |
| No | 63(55.8) | | 45 (53.6) | | 18 (62.1) | 0.34 |
| Yes, in CR1 | 38(33.6) | | 28 (33.3) | | 10 (34.5) |  |
| Yes, others | 12(10.6) | | 11 (13.1) | | 1 (3.4) |  |
|  |  | |  | |  |  |
| **Type of leukemia** | | |  | |  |  |
| ALL | 55(48.7) | | 42 (50.0) | | 13 (44.8) | 0.63 |
| AML | 58(51.3) | | 42 (50.0) | | 16 (55.2) |  |
| **Date of diagnosis** |  | |  | |  |  |
| < 1996/11 | 29(25.7) | | 5 (17.2) | | 24 (28.6) | 0.46 |
| 1996/11- 2001/08 | 28(24.8) | | 7 (24.1) | | 21 (25.0) |  |
| 2001/09- 2007/05 | 28(24.8) | | 7 (24.1) | | 21 (25.0) |  |
| >2007/06 | 28(24.8) | | 10 (34.5) | | 18 (21.4) |  |
| **Irradiation** |  | |  | |  |  |
| CNS | 4(3.5) | | 4 (4.8) | | 0 (0.0) | 0.23 |
| TBI | 3(2.6) | | 3 (3.6) | | 0 (0.0) | 0.30 |
| Testis (only boys) | 3(5.6) | | 3 (8.1) | | 0 (0.0) | 0.23 |

Supplemental Table 3: univariate analysis of the effect of age and the type of leukemia on the occurrence of late sequelae

|  | **Age at diagnosis** | |  | **Type of leukemia** | |  |  |
| --- | --- | --- | --- | --- | --- | --- | --- |
|  | **< 6 months (n = 53)** | **>6 months (n = 60)** | **p** | **ALL (n = 55)** | **AML (n = 58)** | **p** |  |
| **Growth failure** |  |  |  |  |  |  |  |
| major | 19 (35.8%) | 10 (16.7%) | **0.03** | 18 (32.7%) | 11 (19%) | 0.131 |  |
| minor or major | 26 (49.1%) | 28 (46.7%) | 0.852 | 28 (50.9%) | 26 (44.8%) | 0.574 |  |
| GH treatment | 1 (1.9%) | 0 (0%) | 0.469 | 1 (1.8%) | 0 (0%) | 0.487 |  |
| final height of adults (n = 24) | 14 | 10 |  | 12 | 12 |  |  |
| median SDS (range) | -1.1 (-5.08-1.75) | -0.4 (-1.58-0.58) | 0.341 | -0.65 (-5.08-1.08) | -1.01 (-2.08-1.75) | 0.843 |  |
| final height of female adults in cm (n = 13) | 7 | 6 |  | 6 | 7 |  |  |
| median (range) | 158 (140-169) | 159 (155-165) | 0.836 | 159 (140-169) | 158 (157-165) | 0.836 |  |
| final height of male adults in cm (n = 11) | 7 | 4 |  | 6 | 5 |  |  |
| median (range) | 163 (144-185) | 175 (165-178) | 0.412 | 173 (144-181) | 165 (162-185) | 1 |  |
| **Overweight** |  |  |  |  |  |  |  |
| major | 3 (5.7%) | 9 (15%) | 0.134 | 5 (9.1%) | 7 (12.1%) | 0.763 |  |
| minor or major | 7 (13.2%) | 18 (30%) | **0.041** | 13 (23.6%) | 12 (20.7%) | 0.821 |  |
| BMI of adult patients (n = 23) | 13 | 10 |  | 11 | 12 |  |  |
| median (range) | 20.2 (17.04-26.22) | 23.36 (18.31-38.93) | 0.186 | 22.28 (18.31-29.97) | 20.88 (17.04-38.93) | 0.487 |  |
| **Underweight** | 17(32.1%) | 7 (11.7%) | **0.011** | 12 (21.8%) | 12 (20.7%) | 1 |  |
| **Gonadal dysfunction(n = 58)** | 6/26 (23.1%) | 2/32 (6.2%) | 0.123 | 5/31 (16.1%) | 3/27 (11.1%) | 0.712 |  |
| precocious puberty only (n = 58) | 1/26 (3.8%) | 0/32 (0%) | 0.448 | 1/31 (3.2%) | 0/27 (0%) | 1 |  |
| hypogonadism (n = 58) | 6/26 (23.1%) | 2/32 (6.2%) | 0.123 | 5/31 (16.1%) | 3/27 (11.1%) | 0.712 |  |
| girls (n = 27) | 4/11(36.4%) | 2/16 (12.5%) | 0.187 | 3/12 (25%) | 3/15 (20%) | 1 |  |
| boys (n = 31) | 2/15 (13.3%) | 0/16 (0%) | 0.226 | 2/19 (10.5%) | 0/12 (0%) | 0.51 |  |
| **Sex hormone replacement therapy (n = 8)** | 5/6 (83.3%) | 2/2 (100%) | 1 | 4/5 (80%) | 3/3 (100%) | 1 |  |
| **Fertility** | 2 | 1 |  | 1 | 2 |  |  |
| **Hypothyroidism** | 11(20.8%) | 1(1.7%) | **0.001** | 9(16.4%) | 3(5.2%) | 0.069 |  |
| overt hypothyroidism | 2(3.8%) | 0(0%) | 0.218 | 1(1.8%) | 1(1.7%) | 1 |  |
| supplemented hypothyroidism (n=11) | 9/11(81.8%) | 1/1(100%) | 1 | 7/9(77.8%) | 3/3(100%) | 1 |  |
| **Second tumour** | 4(7.5%) | 1(1.7%) | 0.185 | 2(3.6%) | 3(5.5%) | 1 |  |
| thyroid malignancy | 1(1.9%) | 0(0%) | 0.469 | 0(0%) | 1(1.7%) | 1 |  |
| thyroid nodule | 6(11.3%) | 3(5%) | 0.301 | 5(9.1%) | 4(6.9%) | 0.738 |  |
| **Cataract** | 4(7.5%) | 1(1.7%) | 0.185 | 4(7.3%) | 1(1.7%) | 0.198 |  |
| **Cardiac dysfunction** | 6(11.3%) | 3(5%) | 0.301 | 3(5.5%) | 6(10.3%) | 0.491 |  |
| **Viral transmission** | 1(1.9%) | 0(0%) | 0.469 | 0(0%) | 1(1.7%) | 1 |  |
| **Iron overload (n=98)** | 4/46(8.7%) | 1/52(1.9%) | 0.183 | 3/47(6.4%) | 2/51(3.9%) | 0.669 |  |
| **CNS complications** | 4(7.5%) | 2(3.3%) | 0.417 | 3(5.5%) | 3(5.2%) | 1 |  |
| **Alopecia** | 1(1.9%) | 1(1.7%) | 1 | 2(3.6%) | 0(0%) | 0.235 |  |
| **Mean number of sequalae/patient ± SEM** | 1.66 ±0.18 | 1.11±0.12 | **0.011** | 1.49 ±0.17 | 1.24 ±0.14 | 0.248 |  |
| **At least one sequela** | 42(79.2%) | 42(70%) | 0.385 | 42(76.4%) | 42(72.4%) | 0.671 |  |

Statistically significant values are indicated in bold

Supplemental Table 4: univariate analysis of the effect of HSCT on the occurrence of late sequelae

|  | **HSCT** | | | |  |  |  |  |  |  |
| --- | --- | --- | --- | --- | --- | --- | --- | --- | --- | --- |
|  | **No (n = 50)** | **Yes in CR1 (n = 38)** | **Yes others**  **(n = 12)** | **p** |  |  |  |  |  |  |
| **Growth failure** |  |  |  |  |  |  |  |  |  |  |
| major | 14 (22.2%) | 10 (25.6%) | 5 (45.5%) | 0.264 |  |  |  |  |  |  |
| minor or major | 29 (46%) | 19 (48.7%) | 6 (54.5%) | 0.887 |  |  |  |  |  |  |
| GH treatment | 0 (0%) | 1 (2.6%) | 0 (0%) | 0.442 |  |  |  |  |  |  |
| final height of adults (n = 24) | 9 | 13 | 2 |  |  |  |  |  |  |  |
| median SDS (range) | -0.04 (-4.25-1.75) | -0.92 (-5.08-1.08) | -3.11 (-4.14--2.08) | 0.141 |  |  |  |  |  |  |
| final height of female adults in cm  (n = 13) | 4 | 8 | 1 |  |  |  |  |  |  |  |
| median (range) | 160 (155-169) | 158 (156-165) | 140 (140-140) | 0.312 |  |  |  |  |  |  |
| final height of male adults in cm  (n = 11) | 5 | 5 | 1 |  |  |  |  |  |  |  |
| median (range) | 178 (149-185) | 168 (144-181) | 162 (162-162) | 0.603 |  |  |  |  |  |  |
| **Overweight** |  |  |  |  |  |  |  |  |  |  |
| major | 8 (12.7%) | 3 (7.7%) | 1 (9.1%) | 0.812 |  |  |  |  |  |  |
| overweight minor or major | 16 (25.4%) | 8 (20.5%) | 1 (9.1%) | 0.561 |  |  |  |  |  |  |
| BMI of Adult patients(n = 23) | 9 | 13 | 2 |  |  |  |  |  |  |  |
| median (range) | 20.45 (18.31-38.93) | 22.28 (17.04-26.22) | 18.29 | 0.418 |  |  |  |  |  |  |
| **Low weight** | 11(17.5%) | 8(21.1%) | 5 (41.7%) | 0.201 |  |  |  |  |  |  |
| **Gonadal dysfunction( n= 58)** | 1/30 (3.3%) | 5/23 (21.7%) | 2/5 (40%) | **0.027** |  |  |  |  |  |  |
| precocious puberty only (n = 58) | 0/30 (0%) | 1/23 (4.3%) | 0/5 (0%) | 0.483 |  |  |  |  |  |  |
| Hypogonadism (n = 58) | 1/30 (3.3%) | 5/23 (22.7%) | 2/5 (33.3%) | **0.027** |  |  |  |  |  |  |
| girls (n = 27) | 0/12 (0%) | 4/13 (30.8%) | 2/2 (100%) | **0.004** |  |  |  |  |  |  |
| boys (n = 31) | 1/18 (5.6%) | 1/10 (10%) | 0/3 (0%) | 1 |  |  |  |  |  |  |
| **sex hormone replacement therapy**  **(n = 8)** | 1 (100%) | 4/5 (80%) | 2 (100%) | 1 |  |  |  |  |  |  |
| **Fertility** | 2 | 1 |  |  |  |  |  |  |  |  |
| **Hypothyroidism** | 1 (1.6%) | 6 (15.4%) | 3 (27.3%) | **0.002** |  |  |  |  |  |  |
| overt Hypothyroidism | 0 (0%) | 1 (2.6%) | 1 (9.1%) | 0.076 |  |  |  |  |  |  |
| supplemented hypothyroidism (n=11) | 1 (100%) | 6/7 (85.7%) | ¾ (75%) | 1 |  |  |  |  |  |  |
| **Second tumour** | 3 (4.8%) | 1 (2.6%) | 1 (9.1%) | 0.498 |  |  |  |  |  |  |
| thyroid malignancy | 0 (0%) | 0 (0%) | 1 (9.1%) | 0.097 |  |  |  |  |  |  |
| thyroid nodule | 3 (4.8%) | 3 (7.7%) | 3 (27.3%) | 0.056 |  |  |  |  |  |  |
| **Cataract** | 0(0%) | 3 (7.7%) | 2 (18.2%) | **0.01** |  |  |  |  |  |  |
| **Cardiac dysfunction** | 5 (7.9%) | 1 (2.6%) | 3 (27.3%) | **0.046** |  |  |  |  |  |  |
| **Viral transmission** | 0 (0%) | 0 (0%) | 1 (8.3%) | 0.097 |  |  |  |  |  |  |
| **Iron overload(n = 98)** | 1/51 (2%) | 2/37 (5.4%) | 2/10 (20%) | 0.065 |  |  |  |  |  |  |
| **CNS complications** | 2(3.2%) | 1 (2.6%) | 3(27.3%) | **0.017** |  |  |  |  |  |  |
| **Alopecia** | 0 (0%) | 1 (2.6%) | 1(9.1%) | 0.076 |  |  |  |  |  |  |
| **Mean number of sequelae/patient**  **± SEM** | 1.1 ±0.12 | 1.45 ±0.18 | 2.64 ±0.47 | **< 0.001** |  |  |  |  |  |  |
| **At least one sequela** | 45 (71.4%) | 29 (74.4%) | 10 (90.9%) | 0.454 |  |  |  |  |  |  |

Statistically significant values are indicated in bold

Supplemental Table 5: HRQoL of children and adolescents reported by their parents using VSP-Ap

| **overall population** | | | **sex** | | | **type of leukemia** | | | **transplantation** | | | | **age** | | |
| --- | --- | --- | --- | --- | --- | --- | --- | --- | --- | --- | --- | --- | --- | --- | --- |
|  |  |  | **girls** | **boys** |  | **ALL** | **AML** |  | **No** | **yes in CR1** | **yes others** |  | **≤ 6 months** | **> 6 months** |  |
| **VSPAp subscales** | **n** | **mean**  **± SEM** | **mean**  **± SEM** | **mean**  **± SEM** | ***p*** | **mean**  **± SEM** | **mean**  **± SEM** | ***p*** | **mean**  **± SEM** | **Mean**  **± S EM** | **mean**  **± SEM** | ***p*** | **mean**  **± SEM** | **mean**  **± SEM** | ***p*** |
| **Relationship with parents** | 75 | 71.56 ±2.11 | 72.8 ±2.5 | 70.47 ±3.31 | *0.585* | 72.92 ±2.08 | 70.16 ±3.72 | *0.52* | 71.83 ±2.39 | 69.02 ±4.46 | 79.17 ±8.64 | *0.479* | 72.2 ±3.16 | 71.08 ±2.86 | *0.794* |
|  |  |  |  |  |  |  |  |  |  |  |  |  |  |  |  |
| **Body image** | 71 | 75.7 ±3.39 | 67.57 ±5.14 | 84.56 ±3.88 | ***0.01*** | 76.43 ±4.91 | 75 ±4.75 | *0.835* | 81.4 ±3.41 | 67.26 ±7.49 | 66.07 ±14.62 | *0.114* | 68.36 ±6.1 | 81.73 ±3.4 | *0.061* |
|  |  |  |  |  |  |  |  |  |  |  |  |  |  |  |  |
| **Vitality** | 71 | 71.91 ±2.24 | 72.48 ±3.05 | 71.35 ±3.3 | *0.804* | 74.63 ±2.61 | 68.95 ±3.68 | *0.213* | 73.92 ±2.68 | 68.65 ±4.04 | 69.29 ±10.43 | *0.541* | 70.82 ±3.45 | 72.75 ±2.96 | *0.672* |
|  |  |  |  |  |  |  |  |  |  |  |  |  |  |  |  |
| **Relationships with friends** | 67 | 59.02 ±3.06 | 63.57 ±4.12 | 54.87 ±4.42 | *0.157* | 64.07 ±4.06 | 53.83 ±4.47 | *0.094* | 59.86 ±3.88 | 57.65 ±5.35 | 57.5 ±15.12 | *0.941* | 61.01 ±3.3 | 57.6 ±4.72 | *0.555* |
|  |  |  |  |  |  |  |  |  |  |  |  |  |  |  |  |
| **Leisure activities** | 74 | 58.7 ±2.99 | 58.81 ±4.23 | 58.6 ±4.26 | *0.972* | 61.82 ±3.83 | 55.57 ±4.58 | *0.299* | 60.88 ±4.12 | 52.81 ±4.24 | 64.93 ±11.92 | *0.395* | 65.76 ±2.88 | 53.32 ±4.64 | ***0.026*** |
|  |  |  |  |  |  |  |  |  |  |  |  |  |  |  |  |
| **Psychological well-being** | 75 | 76.48 ±2.6 | 75.39 ±3.84 | 77.44 ±3.57 | *0.698* | 80.74 ±3.46 | 71.88 ±3.8 | *0.089* | 77.55 ±3.17 | 76.59 ±4.95 | 69.11 ±10.89 | *0.658* | 73.59 ±4.8 | 78.63 ±2.8 | *0.369* |
|  |  |  |  |  |  |  |  |  |  |  |  |  |  |  |  |
| **Physical well-being** | 77 | 72.48 ±2.42 | 68.58 ±3.46 | 75.91 ±3.34 | *0.132* | 77.08 ±2.92 | 68 ±3.75 | *0.06* | 73.69 ±3.17 | 71.09 ±4.01 | 69.35 ±10.31 | *0.822* | 74.24 ±3.24 | 71.16 ±3.5 | *0.533* |
|  |  |  |  |  |  |  |  |  |  |  |  |  |  |  |  |
| **School work** | 63 | 70.04 ±2.78 | 69.53 ±3.72 | 70.56 ±4.2 | *0.854* | 71.97 ±3.4 | 67.92 ±4.51 | *0.471* | 72.81 ±3.43 | 66.67 ±5.81 | 60 ±4.68 | *0.358* | 71 ±3.51 | 69.41 ±4.02 | *0.782* |
|  |  |  |  |  |  |  |  |  |  |  |  |  |  |  |  |
| **Relationships with teachers** | 64 | 68.36 ±2.78 | 67.36 ±4.42 | 69.24 ±3.54 | *0.739* | 66.8 ±3.79 | 69.92 ±4.1 | *0.578* | 72.46 ±3.28 | 62.27 ±5.35 | 56.67 ±11.61 | *0.126* | 64.58 ±2.92 | 70.63 ±4.07 | *0.233* |
|  |  |  |  |  |  |  |  |  |  |  |  |  |  |  |  |
| **Summary score** | 54 | 69.21 ±2.04 | 66.73 ±2.94 | 71.89 ±2.79 | *0.21* | 72.41 ±2.77 | 66.01 ±2.93 | *0.118* | 72.5 ±2.37 | 63.2 ±4.12 | 62.95 ±7.31 | *0.09* | 66.99 ±2.68 | 70.52 ±2.84 | 0.409 |

Statistically significant values are indicated in bold

Supplemental Table 6: HRQoL of adults using SF-36 questionnaires

| **overall population** | | | **sex** | | | **type of leukemia** | | | **transplantation** | | | | **Age** | | |
| --- | --- | --- | --- | --- | --- | --- | --- | --- | --- | --- | --- | --- | --- | --- | --- |
|  |  |  | **girls** | **boys** |  | **ALL** | **AML** |  | **No** | **yes in CR1** | **yes others** |  | **≤6 months** | **>6 months** |  |
| **Subscales of SF-36** | **n** | **mean ±SEM** | **mean ±SEM** | **mean ±SEM** | ***p*** | **mean ±SEM** | **mean ±SEM** | ***p*** | **mean ±SEM** | **mean ±SEM** | **mean ±SEM** | ***p*** | **mean ±SEM** | **mean ±SEM** | ***p*** |
| **Physical functioning** | 21 | 82.86 ±4.98 | 78.08 ±6.24 | 90.63 ±7.99 | 0.23 | 85 ±5.53 | 80.91 ±8.31 | 0.693 | 78.57 ±10.1 | 90.42 ±4.67 | 52.5 ±17.5 | 0.071 | 82.92 ±6.5 | 82.78 ±8.21 | 0.989 |
|  |  |  |  |  |  |  |  |  |  |  |  |  |  |  |  |
| **Social functioning** | 21 | 73.21 ±5.74 | 72.12 ±7.77 | 75 ±8.84 | 0.814 | 65 ±7.41 | 80.68 ±8.32 | 0.179 | 69.64 ±11.85 | 77.08 ±5.93 | 62.5 ±37.5 | 0.719 | 72.92 ±7.35 | 73.61 ±9.65 | 0.954 |
|  |  |  |  |  |  |  |  |  |  |  |  |  |  |  |  |
| **Role: physical** | 21 | 75.4 ±7.6 | 75.64 ±9.12 | 75 ±14.17 | 0.969 | 70.83 ±9.32 | 79.55 ±12.07 | 0.58 | 65.48 ±14.82 | 85.42 ±7.19 | 50 ±50 | 0.281 | 77.08 ±9.95 | 73.15 ±12.41 | 0.805 |
|  |  |  |  |  |  |  |  |  |  |  |  |  |  |  |  |
| **Role: emotional** | 21 | 53.97 ±7.45 | 56.41 ±10.26 | 50 ±10.91 | 0.687 | 53.33 ±10.18 | 54.55 ±11.26 | 0.938 | 47.62 ±14.29 | 58.33 ±8.33 | 50 ±50 | 0.809 | 55.56 ±10.33 | 51.85 ±11.26 | 0.813 |
|  |  |  |  |  |  |  |  |  |  |  |  |  |  |  |  |
| **Mental health** | 21 | 56.48 ±4.77 | 55.38 ±6.08 | 58.25 ±8.19 | 0.779 | 46.6 ±5.44 | 65.45 ±6.76 | **0.045** | 54.86 ±9.93 | 59.33 ±5.36 | 45 ±25 | 0.694 | 59 ±6.82 | 53.11 ±6.7 | 0.555 |
|  |  |  |  |  |  |  |  |  |  |  |  |  |  |  |  |
| **Vitality** | 21 | 51.07 ±4.46 | 46.73 ±5.11 | 58.13 ±8.07 | 0.224 | 49.75 ±4.78 | 52.27 ±7.56 | 0.786 | 44.64 ±9.74 | 58.33 ±3.67 | 30 ±20 | 0.112 | 55.21 ±5.78 | 45.56 ±6.96 | 0.296 |
|  |  |  |  |  |  |  |  |  |  |  |  |  |  |  |  |
| **Body pain** | 21 | 74.81 ±5.95 | 70.38 ±7.57 | 82 ±9.68 | 0.356 | 70 ±8.97 | 79.18 ±8.07 | 0.455 | 58.29 ±11.28 | 84.33 ±6.34 | 75.5 ±24.5 | 0.131 | 81.58 ±6.93 | 65.78 ±10.02 | 0.196 |
|  |  |  |  |  |  |  |  |  |  |  |  |  |  |  |  |
| **General health** | 21 | 63.1 ±5.98 | 55.62 ±7.64 | 75.25 ±8.48 | 0.113 | 59.6 ±7.89 | 66.27 ±9.15 | 0.591 | 56.71 ±11.25 | 71.75 ±6.98 | 33.5 ±13.5 | 0.142 | 63.83 ±8.05 | 62.11 ±9.48 | 0.891 |
|  |  |  |  |  |  |  |  |  |  |  |  |  |  |  |  |
| **Physical Composite Score** | 21 | 51.92 ±2.43 | 49.51 ±3.24 | 55.84 ±3.39 | 0.214 | 52.27 ±2.8 | 51.6 ±4.02 | 0.896 | 47.68 ±4.44 | 56.08 ±2.8 | 41.8 ±6.45 | 0.111 | 52.78 ±3.18 | 50.78 ±3.96 | 0.695 |
|  |  |  |  |  |  |  |  |  |  |  |  |  |  |  |  |
| **Mental Composite Score** | 21 | 39.56 ±2.57 | 39.68 ±3.44 | 39.37 ±4.08 | 0.955 | 35.85 ±3.31 | 42.93 ±3.73 | 0.176 | 38.66 ±5.2 | 40.6 ±2.93 | 36.46 ±15.37 | 0.884 | 40.53 ±3.65 | 38.27 ±3.72 | 0.676 |

Statistically significant values are indicated in bold.
